# Reversing the Antibiotic Resistance “Yelp Effect” Through the Use of Emotionally Framed Responses to Negative Reviews of Providers: Questionnaire Study

**DOI:** 10.2196/26122

**Published:** 2022-03-22

**Authors:** Monique Mitchell Turner, Hyesun Choung, Quoc-Ha Hannah Mai Bui, Paige Beck, Hera Ashraf

**Affiliations:** 1 College of Communication Arts and Sciences Michigan State University East Lansing, MI United States; 2 Milken Institute School of Public Health George Washington University Washington, DC United States

**Keywords:** online patient review, antimicrobial resistance, emotion, health communication

## Abstract

**Background:**

The overuse of antibiotics has rapidly made antimicrobial resistance a global public health challenge. There is an emerging trend where providers who perceive that their patients expect antibiotics are more likely to prescribe antibiotics unprompted or upon request. Particularly, health care providers have expressed concern that dissatisfied patients will provide disparaging online reviews, therefore threatening the reputation of the practice. To better deal with the negative reviews and inform patients, some health care staff directly respond to patients’ online feedback. Engaging with patients’ online reviews gives providers an opportunity to prevent reputational damage and improve patients’ understanding of the antibiotic resistance problem.

**Objective:**

We aim to test the effectiveness of different response strategies to the negative patient online reviews on the readers’ perceptions of the health care provider and their perceptions related to antibiotics resistance.

**Methods:**

Two experiments were conducted to examine the impact of message tactics (apologizing, inducing fear or guilt) that can be employed by health care providers when responding to patients’ negative online feedback related to not receiving an antibiotic.

**Results:**

Overall, our results demonstrated positive impacts of responding to patients’ online reviews. In study 1, we found apologetic messaging and use of emotional appeals in the response were effective in making readers feel more favorable toward the message. Readers also expressed a greater credibility perception toward the provider and willingness to visit the clinic when emotional appeals were used. Findings from study 2 largely supported the effectiveness of a fear-based response in improving the readers’ credibility perceptions and willingness to visit the clinic. The fear-inducing information was particularly effective among parent readers.

**Conclusions:**

This paper demonstrated that a strategic response to online patient complaints could prevent reputational damage and minimize the potential negative impacts of the review. The results also glean insight into the step toward developing a novel intervention—crafting a persuasive response to patients’ negative feedback that can help improve the understanding of antibiotic resistance problems.

## Introduction

There are at least 2.8 million antibiotic-resistant infections per year in the United States, and as many as 35,000 people die each year due to such infections [1]. Nearly 1 in 3 antibiotics prescribed at outpatient facilities is found to be unnecessary [2]. Reducing the overuse of antibiotics in such facilities, like urgent care centers, can help to lower risks associated with antimicrobial resistance. While the judicious use of antibiotics has been widely promoted, there are still barriers to antibiotic stewardship practices [3]. The most commonly cited reason for unnecessary prescribing is providers’ perception of patient expectation and satisfaction [4,5]. Studies have consistently reported that patient demand greatly influences antibiotic prescribing decisions [6-8]. Likewise, a major determinant of prescribing antibiotics in pediatric care is parents’ expectations and pressure [9,10]. Improving communication between health care providers and patients is suggested as one of the ways to reduce overprescribing [11,12].

This perception of pressure from patients can be exacerbated by other external factors. For example, as patients are increasingly behaving as consumers by posting their experiences with health care providers to websites such as Yelp.com [13], providers may fear that their patient will post a disparaging online review of the clinic and provider when requested antibiotics were not provided. In fact, these felt pressures have become so well known that Wired Magazine termed it “the Yelp effect” [14]. A study showed that 65% of Americans are aware of these online ratings and 37% had avoided a physician based on negative reviews [15]. Anxious over potential negative online reviews, providers might be pressured to comply with unrealistic patient expectations such as prescribing antibiotics even when not warranted.

For the clinics of providers, it is important to engage with patient feedback as those reviews can influence future patients’ visits [16]. For example, if the complaint is about not receiving requested antibiotics during the visit, providers can think of it as an opportunity to improve the patient experience and inform the patient about the potential consequences of taking unnecessary treatments or antibiotics. Nonetheless, few strategies have been tested to assess how they might positively impact patients’ antibiotic perceptions and expectations and ultimately act as a step in stemming the tide of antibiotic resistance. In fact, there are no studies of this ilk in the domain of the so-called Yelp effect, yet there are effective risk and health communication strategies that should be tested in this domain. Scholars have long proposed that in order to garner attention, increase message scrutiny, and change attitudes, emotional appeals can be useful [17,18]. Two emotional appeals that have received attention are fear and guilt.

Fear appeals, which communicate an impending threat coupled with behavioral recommendations to mitigate the threat, have consistently been shown to effectively change attitudes [19]. If the fear appeal effectively communicates the threat and receivers believe the response is an effective measure they can take, then message receivers judge that the risk is dangerous and can affect their lives. A meta-analysis investigated fear appeal’s effectiveness for influencing attitudes, intentions, or behaviors and found that fear appeals are effective [20]. In addition, practitioners find that fear appeals can be effectively used to persuade audiences when used appropriately [21]. When communicating antibiotic resistance, fear can be aroused by emphasizing the potential harm or danger that will befall individuals if they take unnecessary antibiotics. In this case, we argue that fear messaging can increase the perception of antibiotic resistance risk. Thus, the reader of a negative review would believe that a provider who did not provide antibiotics was being careful and protecting the patient. This could mitigate unfavorable effects of the negative review.

H1: A response from the clinic that features fear-inducing antibiotic resistance information will have a positive impact on (a) message favorability, (b) provider credibility, and (c) increased willingness to visit the clinic in the future relative to no response or a simple apology (ie, apologetic response).

Guilt appeals, though, work through a different mechanism. Guilt appeals communicate that a harm could be potentially caused by our own actions or inactions and are commonly used in health and risk communication [22-25]. These subtle messages remind audiences of their moral code (eg, do not harm others) and can spark both attitude change and behavior change. Turner’s research [22,26,27] revealed that when messages convey a discrepancy between individuals’ moral norms and their potential future behavior, they are less likely to engage in the harmful behavior. In this case, people would be reminded that pressuring doctors for antibiotics ultimately affects the antibiotic health crisis. Guilt is seen as a prototypical moral emotion, and it is repeatedly used in persuasion campaigns for its behavioral consequences [24].

H2: A response from the clinic that features guilt-inducing antibiotic resistance information will have a positive impact on (a) making readers feel favorable toward the message, (b) increasing the credibility of the reviewed provider, and (c) increase the willingness to visit the clinic in the future relative to no response or an apologetic response.

As indicated in hypotheses 1 and 2, one type of baseline comparison group we test is a simple apology. Apologies are considered to be the essential response component that service providers can use to handle consumer complaints [28,29]. Health care providers can engage in complaints by apologizing and taking responsibility for patients’ negative experiences. This can help them to reclaim a favorable impression as prior research has documented that apologies are effective for reestablishing trust [30,31]. Accordingly, we hypothesized the following:

H3: A response from the clinic apologizing for patients’ negative experience will have a positive impact on (a) making readers feel favorable toward the message, (b) increasing the credibility of the reviewed provider, and (c) increasing the willingness to visit the clinic in the future relative to providing no response to the review.

## Study 1

### Introduction

Based on the vast literature on emotionally framed health and risk communication, we tested strategic communication regarding antibiotic resistance in the form of replies to negative patient online reviews. We test the effectiveness of fear, guilt, and apologetic message strategies used when responding to a patient’s negative online reviews complaining about not receiving requested antibiotics during its visit to the local urgent care. We accomplish these objectives by conducting a randomized controlled experiment with the goal of examining the impact of different response message strategies on readers’ perceptions of the health care providers and perceptions and knowledge about antibiotics resistance.

### Methods

A between-subjects experimental design with 4 message conditions—control (no response), apology messaging, fear-based messaging, and guilt-based messaging—was used. Participants were asked to imagine that they have moved to a new town and need to find a local urgent care. Then, participants viewed a review site (like Yelp, but deidentified) where a patient provides a negative review of an urgent care. The patient arrived at urgent care with a cold and a sore throat but did not receive an antibiotic. The wording of the fictitious online review was based on real online reviews. Participants also saw the urgent care clinic representative’s (staff or care provider) response to the negative review based on the experimental condition. See Multimedia Appendix 1 for stimulus. Participants were allowed to view this page as long as they needed to. Afterward, participants were asked to complete a short survey and asked their favorability toward the response; the credibility of the clinic, provider and staff; and willingness to visit the clinic in the future (see Multimedia Appendix 2). Participants were recruited through the Amazon Mechanical Turk platform. A total of 216 adults (55% (119/216) male, 81% (175/216) White) from around the nation were paid to take part in a 15-minute experiment. About 64% (138/216) of the participants were aged 30 to 49 years, and 42% (91/216) had a Bachelor’s degree.

### Results

To test the effect of response strategies on outcome variables, we ran a series of 1-way analyses of variance (ANOVAs) with Tukey post hoc testing. There was a statistically significant difference in readers’ favorability toward the message (F_3, 212_=29.81, P<.001), perceived provider credibility (F_3, 212_=3.66, P=.01), and willingness to visit the reviewed clinic F_3, 212_=4.01, P=.008) across message conditions (see Table 1).

**Table 1:** Effects of response strategies by messaging condition in study 1 (Note: Means in a row sharing superscripts are significantly different from one another).

Variable	Control (1)		Apology (2)			Fear (3)			Guilt (4)			η^2^
	Mean	SD	Mean	SD	Mean		SD	Mean		SD		
Favorability toward the message	2.90^*^	0.78	3.86^#^	0.84	4.44^^^		0.89	4.30^^^		1.00	.297	
Provider credibility perception	3.65^*^	1.17	3.71^*^	1.08	4.20^#^		1.06	4.16^*,#^		1.11	.049	
Willingness to visit the clinic	3.56^*,#^	0.98	3.36^*,#^	0.98	3.89^*,^^		1.19	3.98^*,^^		1.08	.054	

These data support the hypothesis that a fear-inducing message was effective in making readers feel more favorable toward the response (mean 4.44 [SD .89]) compared to the control group (P<.001). This response was also more effective than an apology message (mean 3.86 [SD .84], P=.003). H1a was supported. Furthermore, the fear message increased readers’ credibility perception toward the reviewed provider (mean 4.20 [SD 1.06]) compared to the control group (mean 3.65 [SD 1.17], P=.06), and the apology message condition (mean 3.71 [SD 1.08], P=.09). H1b was supported. Respondents’ willingness to visit the clinic in the future was only significantly different between the fear (mean 3.89 [SD 1.19]) condition and the apology condition (mean 3.36 [SD .98], P=.04). The mean difference between the fear-inducing condition and the control condition was not significant. Accordingly, H1c was partially supported.

Similarly, guilt-inducing messaging made readers feel more favorable toward the response (mean 4.30 [SD 1.00]) relative to apologetic messaging (mean 3.86 [SD .84], P=.047). Thus, H2a was supported. The guilt message did not increase readers’ credibility perception toward the provider (H2b not supported). When a response offered guilt-based antibiotic resistant information, readers were more willing to visit the clinic in the future (mean 3.98 [SD 1.08]) compared to the apology message condition (mean 3.36 [SD .98], P=.01). Nonetheless, a statistically significant difference was not observed when the guilt condition was compared to the control condition (mean 3.56 [SD .98]). Therefore, H2c was partially supported.

We found that the readers who read an apology message (mean 3.86 [SD .84]) from a clinic, which received a negative review from a past patient, felt more favorable toward the response compared to the readers who read the negative patient review with no reply from the clinic (mean 2.90 [SD .78], P<.001). However, there was no significant effect of apology messaging on the credibility perception and willingness to visit the clinic. Thus, H3a was supported, but H3b and H3c were not supported.

The findings from study 1 provide empirical support for the idea that engaging with patients’ negative reviews with an effective and evidence-based communication strategy can prevent potential reputational damage and improve the patient-provider relationship. In doing so, our findings suggest that rather than just apologizing for patients’ negative experiences (which implies admitting blame), using emotional appeals would further enhance the effectiveness of the message in building favorable attitudes, credibility, and increasing potential patients’ chance to visit the clinic in the future.

Specifically, we found that apologizing for a patient’s negative experience can make readers feel more favorable, but it did not increase credibility perceptions or willingness to visit the clinic. If the goal of the communication is to restore credibility and increase patient visits, emotion-based messaging is more effective than not responding or apologizing. We found the fear-inducing information related to antibiotic resistance was particularly effective in increasing the credibility perception of the provider.

These findings can help empower managers, owners, and providers within urgent care clinics to deal with the pressures patients might exert when they want antibiotics. It is critical that providers feel confident that they can engage in clinically sound practices without harming their, or the clinic’s, credibility. It is critical to assess the replicability of these findings, though, so we replicated and extended this study with a second national panel of participants.

## Study 2

### Introduction

A second study was conducted to test the robustness of some of the key findings from the first study. In a pediatric care setting, parent pressure to prescribe antibiotics for their child is a barrier for working toward a strict adherence of antibiotic prescription [9,10]. Thus, in the second experiment, we test the effectiveness of response strategies when a negative online review is left by a parent who did not get antibiotics prescribed for their sick child. In study 2, we also compare which type of message strategy (control vs apology vs fear vs guilt) is more effective in altering readers’ antibiotics expectations and perceptions.

In study 1, we examined if strategic messaging could improve readers’ perceptions toward the reviewed provider. To test the robustness of the findings, we suggest the following hypotheses:

H1: A response from the clinic that features fear-inducing antibiotic resistance information will increase (a) the credibility of the reviewed provider and (b) the willingness to visit the clinic in the future relative to providing no response or an apologetic response.

H2: A response from the clinic that features guilt-inducing antibiotic resistance information will increase (a) the credibility of the reviewed provider and (b) the willingness to visit the clinic in the future relative to providing no response or an apologetic response.

H3: A response from the clinic apologizing for patients’ negative experience will increase (a) the credibility of the reviewed provider and (b) the willingness to visit the clinic in the future relative to providing no response to the review.

In study 2, we also test if the clinic’s response can be an effective educational intervention that improves people’s understanding of the antibiotic resistance problem. We predict that an effective message will lower people’s unrealistic expectations for antibiotics and reduce the conception of better to be safe than sorry (BSTS). The latter, BSTS, is the common misperception that even in the cases when taking antibiotics are unlikely to be effective; patients perceive that there is some chance that antibiotics might be effective and have little risk. Patients prefer to take antibiotics since doing so provides the possibility of getting better, and it is often the patients’ rationale for requesting antibiotics [32]. Thus, we try to answer the following question:

RQ1: Which response message strategy (apology vs fear-inducing vs guilt-inducing messaging) is more effective in (a) lowering people’s antibiotics expectations and (b) reducing BSTS misconception compared to the control group condition?

As the online review used in study 2 features a parent’s experience with a clinic, we also evaluate whether there is any difference in the effectiveness of the response strategies between parent readers and nonparent readers with the following research questions.

RQ2: Is there any difference in the level of (a) credibility perception, (b) willingness to visit, (c) antibiotics expectations, and d) BSTS misconception between parent readers and nonparent readers?

RQ3: Is there any difference in the effects of response strategies on the (a) credibility perception, (b) willingness to visit, (c) antibiotics expectations, and (d) BSTS misconception while controlling for the parental status?

### Methods

#### Study Overview

A 4 (message type: control, apology, fear-based, guilt-based ) × 2 (parental status: parent vs nonparent) between-subjects experimental design was used. Multimedia Appendix 1 includes the stimulus used in the study. Participants aged 18 years and older and living in the United States were recruited. As with study 1, we used the MTurk survey platform for this 10-minute experimental survey. A total of 400 US adults took the survey; after removing incomplete responses, a total of 390 responses were used in the analysis. The median age group of participants was 30 to 49 years, and 62% (242/390) of participants were male. About 58% (226/390) of participants identified their race as White and 58% (226/390) had a Bachelor’s degree. A summary of survey measurements is presented in Multimedia Appendix 2.

#### Data Analysis

A 2-way ANOVA was conducted to determine the effects of message strategies and parental status for each outcome variable. Table 2 reports a summary of test results. An additional simple effect analysis was conducted to probe interaction effects.

**Table 2:** Summary table for 2-way analysis of variance of the effects of messaging strategies and parental status in study 2.

Variable and source		MS^a^	F score	P value	η_p_^2^
**Provider credibility perception**					
	Message^b^	5.96	4.02	.008	.031
	Parental status^c^	33.24	22.45	<.001	.056
	Message × parental status^b^	3.19	2.15	.093	.017
**Willingness to visit the clinic**					
	Message^b^	8.23	19.36	<.001	.033
	Parental status^c^	36.57	19.36	<.001	.048
	Message × parental status^b^	7.09	3.75	.011	.029
**Antibiotic expectation**					
	Message^b^	0.49	0.56	.640	.004
	Parental status^c^	2.34	2.7	.102	.007
	Message × parental status^b^	0.625	0.72	.541	.006
**BSTS^d^ misconception**					
	Message^b^	1.13	1.28	.281	.010
	Parental status^c^	24.66	27.99	<.001	.068
	Message × parental status^b^	0.606	0.69	.560	.005

^a^MS: mean squares.

^b^*df* = 3, 382.

^c^*df* = 1, 382.

^d^BSTS: better safe than sorry.

### Results

To test H1-H3, we first compared the mean scores of credibility perception and willingness to visit the clinic across the 4 message conditions (Table 3). We found that response strategies have statistically significant effects on readers’ credibility perception of the provider and willingness to visit the clinic (see Table 2).

**Table 3:** Effects of response strategies by messaging condition in study 2 (Note: Means in a row sharing superscripts are significantly different from one another).

Variable	Control (1)			Apology (2)			Fear (3)			Guilt (4)			η^2^
	Mean	SD	Mean		SD	Mean		SD	Mean		SD		
Provider credibility perception	2.51^*^	1.13	2.92^*,#^		1.27	3.09^#,^^		1.29	2.82^*,#,^^		1.3	.028	
Willingness to visit the clinic	2.61^*^	1.30	2.94^*,#^		1.48	3.26^#,^^		1.42	3.05^*,#,^^		1.5	.027	
Antibiotic expectation	3.24^*^	0.94	3.25^*^		0.90	3.11^*^		0.92	3.15^*^		0.97	.004	
BSTS misconception	2.91^*^	0.95	2.71^*^		0.96	2.76^*^		0.95	2.71^*^		1.01	.008	

These data support the hypothesis that fear-inducing information can increase providers’ credibility perception (mean 3.09 [SD 1.29], P=.006) and willingness to visit the clinic (mean 3.26 [SD 1.42], P=.008) when compared to the control group (credibility: mean 2.51 [SD 1.13]; willingness to visit: mean 2.61 [SD 1.30]), but the differences were not statistically significant compared to the apology message condition (mean 2.98 [SD1.27]). Thus, H1 was partially supported. The guilt-inducing response did not increase credibility perception. Study 1 showed that the guilt message can increase readers’ willingness to visit when compared to the apology condition. However, in study 2, which is in the pediatric context, the effect was not statistically significant. Accordingly, H2 was not supported. Regarding H3, there were no statistically significant effects of the apology message on the credibility perception or willingness to visit the clinic (H3 not supported), repeating the findings from study 1.

To answer RQ1, we compared the mean scores of the expectations for antibiotics and BSTS misconception (see Table 3) across the message conditions. We found no significant main effects of message strategies on antibiotic expectation and BSTS misconception.

Two participant groups (parents vs nonparents) were compared to answer RQ2 (see Table 4). Participants’ parental status had a statistically significant impact on provider credibility perception, willingness to visit the clinic, and the BSTS misconception. Parents perceived the provider less credible (mean 2.56 [SD 1.19], P<.001) compared to nonparents (mean 3.13 [SD 1.29]). Parents were also less willing to visit the clinic in the future (mean 2.69 [SD1.42], P<.001) than nonparents (mean 3.26 [SD 1.40]). Parent readers exhibited greater BSTS misconception (mean 3.01 [SD .86], P<.001) than nonparent participants (mean 2.52 [SD 1.01]). There was no statistically significant difference observed in their expectations of getting antibiotics.

**Figure 1:**
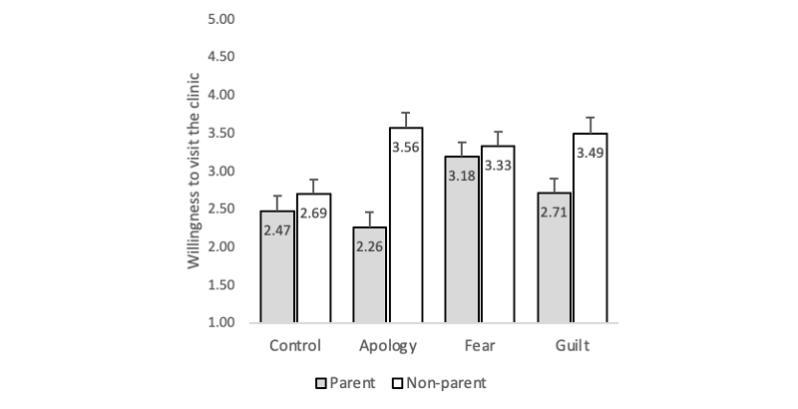
Interaction effects between message condition and parental status on willingness to visit the clinic in Study 2.

Finally, a statistically significant interaction effect between parental status and the message strategy was detected on individuals’ willingness to visit the clinic in the future (RQ3, see Figure 1). For the parent participants (F_3, 384_=3.51, P=.02), the response that elicited fear increased their willingness to visit the clinic (mean 3.18 [SD .20]) significantly more than the control (mean 2.52 [SD .20], P=.02) and the apology (mean 2.31 [SD .20], P=.002) message conditions. For the nonparent participants (F_3, 384_=3.93, P=.009), all 3 types of response messages increased their willingness to visit the clinic (apology: mean 3.56 [SD .20], P=.002; fear: mean 3.33 [SD .19], P=.02; guilt: mean 3.49 [SD .21], P=.006) when compared to the control group condition (mean 2.69 [SD .20]). There was no statistically significant difference among the three message conditions for nonparents.

**Table 4:** Mean and standard deviations for effects of parental status in study 2.

Variable	Parent			Nonparent			η^2^		P value
	Mean	SD	Mean		SD				
Provider credibility perception	2.56	1.18	3.13		1.29	.051		<.001	
Willingness to visit the clinic	2.69	1.42	3.26		1.40	.040		<.001	
Antibiotic expectation	3.26	0.97	3.11		0.88	.007		.11	
BSTS^a^ misconception	3.01	0.86	2.52		1.01	.065		<.001	

^a^BSTS: better safe than sorry.

## Discussion

### Principal Findings

Emotional appeals can be used to better communicate the information about the risks and consequences of inappropriate use of antibiotics. Appealing to human emotion has long been known to help garner attention, engage message receivers cognitively, and change beliefs and intentions [18]. This study showed that such emotion-based tactics need not be solely used in public service announcements; rather, such known message strategies can also be used in responses to online reviews. Providers and clinic owners can and should communicate good clinical practice whenever they can do so. Instead of being worried about negative online reviews, it is possible that these moments can be leveraged as teachable moments, and we believe this paper provides insight into how to develop evidence-based online interventions [33,34].

Study 1 was conducted in the primary care context. This study revealed that fear-based messaging can increase message favorability, provider credibility, and willingness to go to the clinic compared to a simple apology. Given that the apologetic response is the most common response to negative online reviews, this information is critical for clinics. These results can be explained by the studies that showed that when communication of the severity of a health threat is combined with communication of susceptibility to the threat, message receivers are more likely to engage with the message content [17]. Particularly if the message communicates a solution (ie, efficacy), message receivers will engage in a process known as danger control whereby they are fearful of the health threat, understand the threat can be averted, and want to engage in behaviors that would control and prevent it [35].

The findings from study 2 further demonstrated the effectiveness of using a fear-frame when responding to a patient’s negative online review. Another important finding was that while different message strategies were equally effective in increasing nonparents’ willingness to visit, only the fear-inducing message had an impact on parent readers. Thus, when responding to a pediatrics online review often left and read by parents, fear messaging, demarked by communicating susceptibility to, and severity of a risk, can help to mitigate potential negative consequences of a negative online review.

Moreover, we found that parents were overall more influenced by the negative online review than nonparents. Parents perceived the provider as less credible, and they were less willing to visit the clinic than nonparents. This could be because parents are generally concerned about doing the right thing for their child. When it comes to medical decisions, they experience greater uncertainty and anxiety with their decisions for their ill child [36]. Parents held greater BSTS misperception (taking antibiotics has little risk associated and increases the chance of getting better even when it might not be needed) because they are more worried about missing an opportunity to treat their child’s serious illness than a long-term side effects of antibiotics.

Finally, the emotion-based response strategies did not have statistically significant effects on lowering individuals’ antibiotic expectations or correcting the BSTS misperception. Although we found limited educational effects of the response messages, as changing patients’ perception is critical to reduce the overprescribing of antibiotics, future studies should examine the effects of different message strategies and interventions that can improve people’s understanding of antibiotic resistance.

To this point, it is critical that expertise in messaging strategies is sought before clinicians design responses to online reviews (or develop message strategies generally). It is well known that certain messaging strategies can fail when used with the wrong audience or in the wrong context. In this study, we found that while nonparent readers were receptive to various messaging strategies, only fear-inducing messaging was effective among parent readers. Previous studies reported that a persuasive appeal’s effectiveness varies by the audience, and aligning message strategies with the recipient’s personal traits and emotional status can increase the message’s impact [37,38]. Likewise, certain messaging strategies can fail when used with the wrong audience or in the wrong context [39]. Thus, practitioners should critically analyze their target audience and develop the best messaging strategies accordingly.

### Limitations

There are limitations to this research. Certainly, these studies did not use probability-based samples (although they did use random assignment), and thus, we should be cautious when generalizing the findings. Additionally, this study only examined one iteration of fear or guilt; but guilt and fear messages vary a great deal in intensity. Overly intense messages can cause anger and perceptions of being manipulated—so such strategies must be used subtly and thoughtfully. We were also mainly interested in the effects of messages in both studies, but the tested outcome variables (ie, favorability, credibility perception, and willingness to visit) are interrelated. For instance, it is possible that the favorability or credibility perception may predict readers’ desire to visit the clinic. Thus, future studies could further our findings by investigating a causal model among the outcome variables engaging in mediation analysis. Finally, it may be that clinics are uncomfortable replying to negative online reviews at all. Although we hope that these data provide confidence that replying is a good idea, we recognize that some clinic managers will remain reticent to do so. In those cases, the emotional appeals here could be used to inform in-clinic messages that might change or adjust expectations prior to a negative review happening.

### Conclusion

It is imperative that providers feel empowered to follow clinically sound practices. In this case, that means not feeling pressured to prescribe antibiotics when they are not needed. Health communication theories can provide strategies that can be used to assist in these efforts. For example, some clinics have tested the impacts of displaying posters using a commitment strategy [40], as a well-known strategy in communication and psychology, on changing patient expectations [41]. Overall, the strategy has shown promising results in affecting patient perceptions and expectations. Here, we examined the role of emotionally framed messages. Health communication scholars have tested emotional appeals on attitude change for nearly a century, finding that they can have a positive impact at how people perceive health risks. In this case, the key outcomes were to increase the credibility of a provider and willingness to visit a clinic. More studies of this ilk must be conducted going forward so that we can arm providers with efficacious communication devices that help them do their job.

## Appendix

Multimedia Appendix 1 Examples of stimuli.

Multimedia Appendix 2 Summary survey questionnaires and reliability coefficients.

## Abbreviations

ANOVA: analysis of variance
BSTS: better to be safe than sorry
