# Supplementary material for: Reversing the Antibiotic Resistance “Yelp Effect” Through the Use of Emotionally Framed Responses to Negative Reviews of Providers: Questionnaire Study
Source: JMIR Form Res. 2022 Mar 22;6(3):e26122. doi: 10.2196/26122 (PMC8984826; doi:10.2196/26122)

**Multimedia Appendix 1.** Examples of stimuli.

The first stimulus represents the self perspective from Study 1. The second stimulus represents the parent perspective from Study 2. Both responses are fear responses. For additional stimuli, contact the first author.


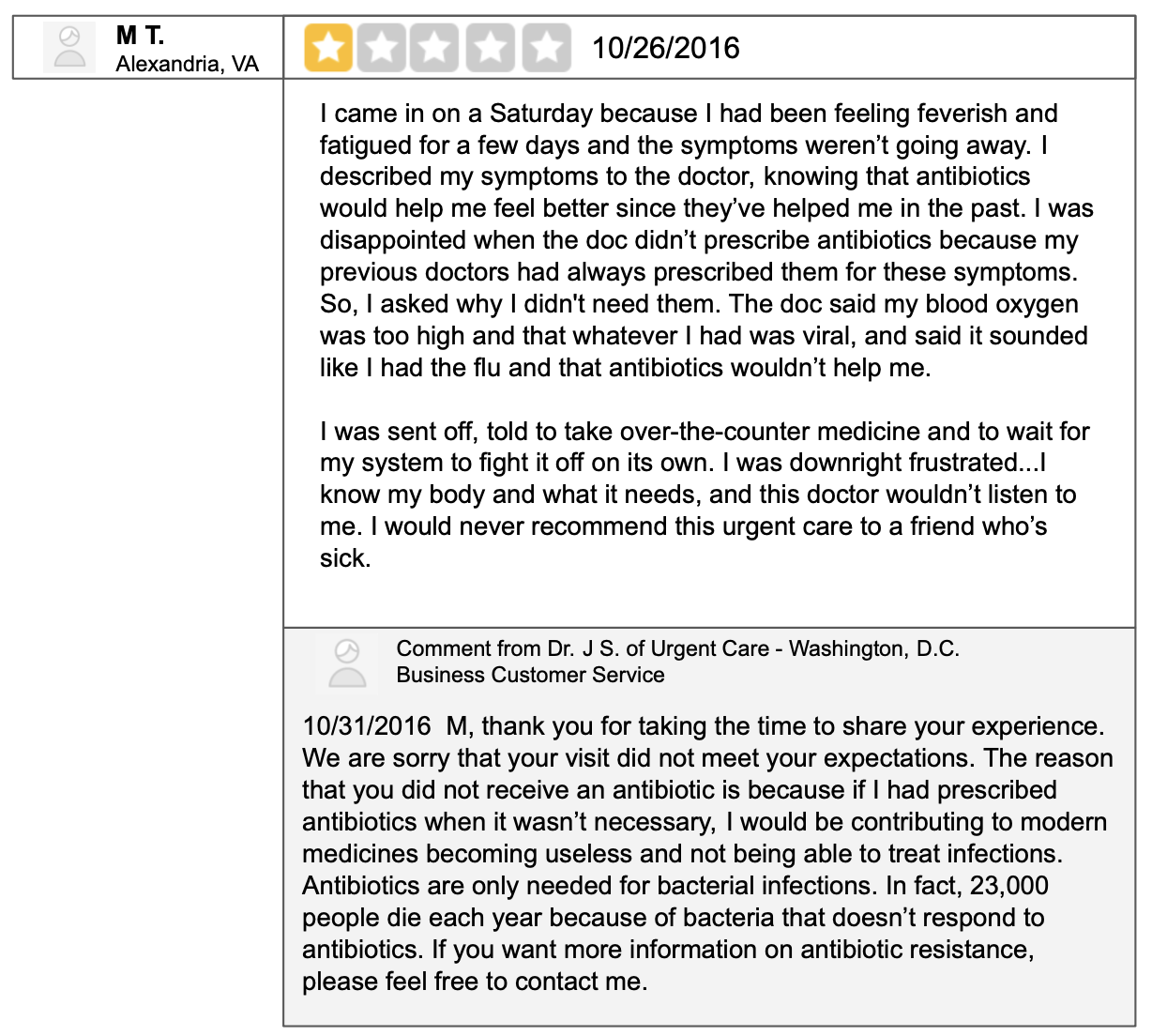


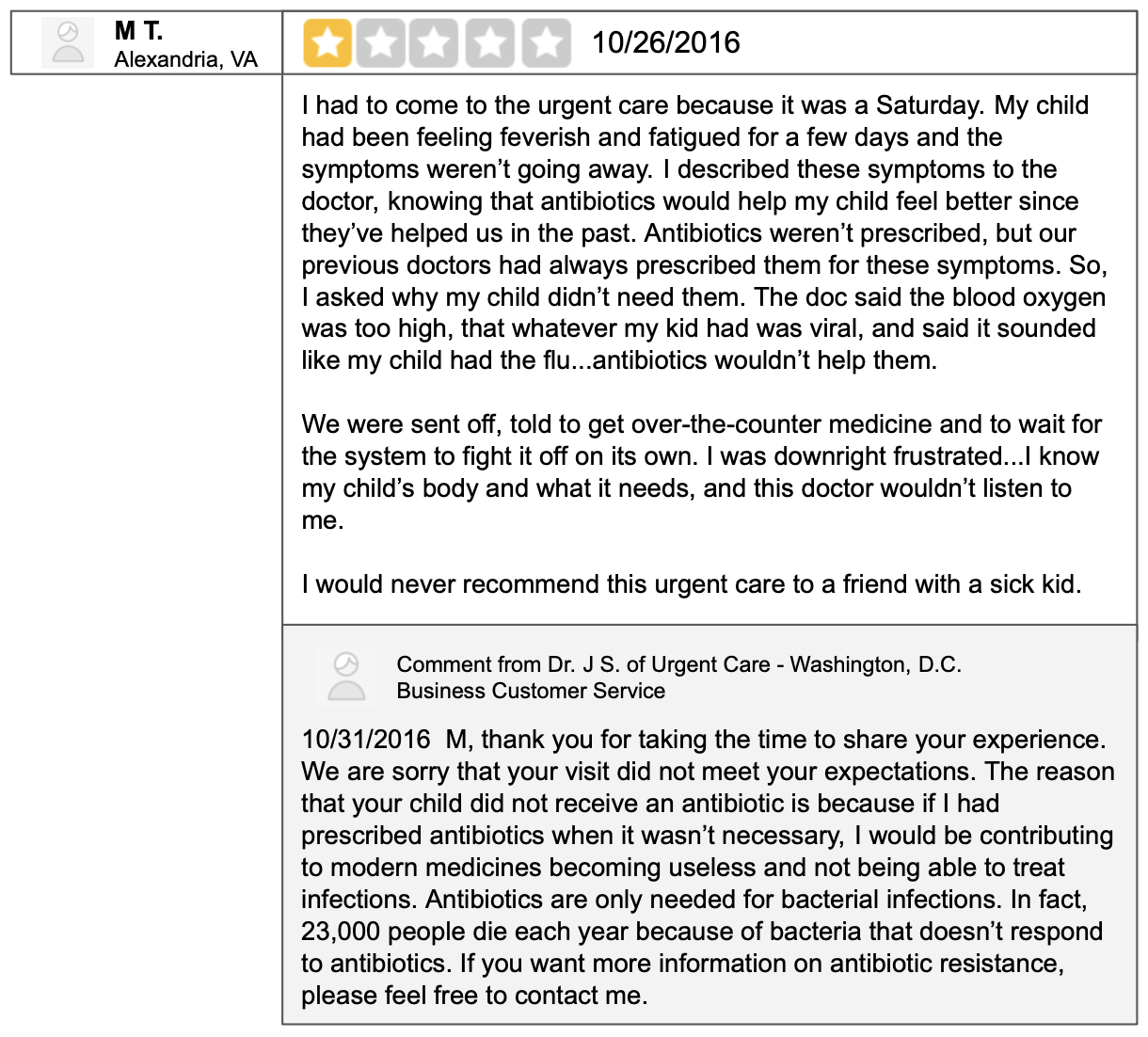

Supplement: Multimedia Appendix 1 [file formative_v6i3e26122_app1.docx]
