# Supplementary material for: Reversing the Antibiotic Resistance “Yelp Effect” Through the Use of Emotionally Framed Responses to Negative Reviews of Providers: Questionnaire Study
Source: JMIR Form Res. 2022 Mar 22;6(3):e26122. doi: 10.2196/26122 (PMC8984826; doi:10.2196/26122)
Supplement: Multimedia Appendix 2 [file formative_v6i3e26122_app2.docx]

**Multimedia Appendix 2.** Summary survey questionnaires and reliability coefficients.

| Variable | Survey items | Scale | Mean (SD) | Reliability |
| --- | --- | --- | --- | --- |
| **Study 1** |  |  |  |  |
| Favorability toward the message | I like that the clinic responded to the review. | 1 (strongly disagree) – 5 (strongly agree) | 4.05 (1.01) | Spearman-Brown coefficient = .857 |
|  | I liked the clinic’s response to the review. |  |  |  |
| Provider credibility perception | I question the credibility of the doctor mentioned in this review. (Reversed) | 1 (strongly disagree) – 5 (strongly agree) | 4.02 (1.10) | Spearman-Brown coefficient = .927 |
|  | I would not want to be treated by this doctor. (Reversed) |  |  |  |
| Willingness to visit the clinic | I would never go to this clinic, based on what I've read. (Reversed) | 1 (strongly disagree) – 5 (strongly agree) | 3.82 (1.08) | Spearman-Brown coefficient = .897 |
|  | Based on what I read, I would go to this clinic. |  |  |  |
| **Study 2** |  |  |  |  |
| Antibiotic expectation | Ear pain | 1 (low expectation) – 5 (high expectation) | 3.19 (1.67) | Cronbach’s alpha = .868 |
|  | Deep cut |  |  |  |
|  | Sore throat |  |  |  |
|  | Fever |  |  |  |
|  | Cough |  |  |  |
|  | Stomachache |  |  |  |
|  | Sinus pressure |  |  |  |
|  | Strep throat |  |  |  |
| BSTS misconception | I don’t know if an antibiotic can make me better, but it can’t hurt to take them. | 1 (strongly disagree) – 5 (strongly agree) | 2.77 (1.68) | Cronbach’s alpha = .803 |
|  | I should take antibiotics because it’s better to be safe than sorry. |  |  |  |
|  | I should not take antibiotics if they might not make me better. (Reverse) |  |  |  |
|  | Antibiotics do not have harmful side effects. |  |  |  |
|  | I won’t get better unless I take antibiotics. |  |  |  |
| Provider credibility perception | I question the credibility of the doctor mentioned in this review. (Reversed) | 1 (strongly disagree) – 5 (strongly agree) | 2.84 (1.27) | Spearman-Brown coefficient = .781 |
|  | I would not want to be treated by this doctor. (Reversed) |  |  |  |
| Willingness to visit the clinic | I would never go to this clinic, based on what I've read. (Reversed) | 1 (strongly disagree) – 5 (strongly agree) | 2.97 (1.44) | N/A |
